# A comprehensive review on tyrosinase inhibitors

**DOI:** 10.1080/14756366.2018.1545767

**Published:** 2019-01-03

**Authors:** Samaneh Zolghadri, Asieh Bahrami, Mahmud Tareq Hassan Khan, J. Munoz-Munoz, F. Garcia-Molina, F. Garcia-Canovas, Ali Akbar Saboury

**Affiliations:** a Department of Biology, Jahrom Branch, Islamic Azad University, Jahrom, Iran;; b Aura Dynamics, Tromsø, Norway;; c Group of Microbiology, Department of Applied Sciences, Northumbria University at Newcastle, Newcastle Upon Tyne, UK;; d GENZ-Group of Research on Enzymology, Department of Biochemistry and Molecular Biology-A, Regional Campus of International Excellence "Campus Mare Nostrum", University of Murcia, Espinardo, Murcia, Spain;; e Institute of Biochemistry and Biophysics, University of Tehran, Tehran, Iran

**Keywords:** Tyrosinase, inhibitor, depigmentation agents, antibrowning compounds

## Abstract

Tyrosinase is a multi-copper enzyme which is widely distributed in different organisms and plays an important role in the melanogenesis and enzymatic browning. Therefore, its inhibitors can be attractive in cosmetics and medicinal industries as depigmentation agents and also in food and agriculture industries as antibrowning compounds. For this purpose, many natural, semi-synthetic and synthetic inhibitors have been developed by different screening methods to date. This review has focused on the tyrosinase inhibitors discovered from all sources and biochemically characterised in the last four decades.

## Introduction

Browning of fruits, fungi and vegetables and hyperpigmentation in human skin are two common undesirable phenomena. Tyrosinase is the main enzyme recognised as responsible for this enzymatic browning and melanogenesis in mammals^1^^,^^2^. This encouraged researchers and scientists to focus on the identification, isolation, synthesis and characterisation of new potent tyrosinase inhibitors for various application in the food^3^, cosmetics^4^ and medicinal industries. However, very few inhibitors are qualified for clinical use and skin-whitening agents. Moreover, as the clinical and industrial demands for tyrosinase inhibitors increase, *in vitro* assays and improved screening techniques are also undergoing rapid development for *in vitro* high-throughput screening tyrosinase inhibitors and putative skin-whitening agents^5^. In other words, sensitive and correct assay methods for screening and development of effective tyrosinase inhibitors are of great importance. For this purpose, several spectrophotometric^6–10^, chromatographic^11–17^, electrophoretic^18–22^, radiometric^23^^,^^24^ and electrochemical^25–27^ assays have been applied and developed by researchers so far. Recently, a novel fluorescent biosensor^28^ and tyrosinase-based thin-layer chromatography-autography have been suggested for tyrosinase inhibitor screening^29^.

Additionally, further improvements of *in vitro* detection methods for rapidly screening tyrosinase inhibitors may be achieved through using virtual screening^30^ and construction of quantitative structure–activity relationship (QSAR) models of inhibitors^31^^,^^32^. Thus, a combination of bioinformatics simulation and biological *in vitro* analysis will be useful to understand the functional mechanisms of the tested compounds^9^^,^^21^^,^^27^^,^^33–48^. Lately, Gao et al. have performed a virtual screening from Traditional Chinese medicine (TCM) and predicted tyrosinase inhibition by 3 D QSAR pharmacophore models^49^. For more information about successful utilisation of computational tools like QSAR-based and ligand-based virtual screening, a review published by Khan in 2012 organised and summarised novel and potent inhibitors of the enzyme^50^. Furthermore, with regard to tyrosinase inhibition importance, several other reviews have presented the organisation of tyrosinase inhibitors from natural, semi- and full synthetic sources^1^^,^^51–62^.

The present review also focuses on the tyrosinase inhibitors discovered from all sources, including synthetic compounds, extracts and active ingredients of natural products, virtual screening and structure-based molecular docking studies published in the last four decades. We hope that the knowledge offered in this review serves as an updated comprehensive database contributing to the development of new safe and efficient anti-tyrosinase agents for the prevention of browning in plant-derived foods, seafood and hyperpigmentation treatments.

## The role of tyrosinase in the melanin biosynthesis

**Figure 1:**
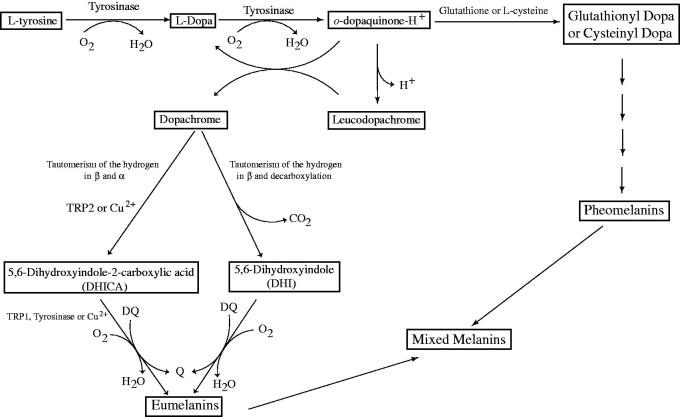
Scheme of the biosynthetic pathway of eumelanins and pheomelanins. The activities of tyrosinase are indicated in the scheme. Moreover, the enzyme can oxidize DHICA to its *o*-quinone directly, or it can oxidize DHICA and DHI indirectly via the formation of *o*-dopaquinone. TRP2 (dopachrome tautomerase) or Cu^2+^ can participate in the evolution of dopachrome to DHICA. The oxidation of DHICA can be catalyzed by TRP1, (DHICA oxidase), tyrosinase or Cu^2+^. When glutathione or L-cysteine attack *o-*dopaquinone, glutathione-dopa or cysteinyl-dopa adducts are formed and these later evolve to pheomelanins ^67^.

Melanins, the main pigment primarily responsible in the skin, hair and eyes pigmentation of human, are produced by melanocytes through melanogenesis. Melanogenesis and skin pigmentation are the most important photoprotective factor in response to ultraviolet radiation damaging from the sun and skin photo-carcinogenesis. The abnormal loss of melanin and depigmentation can be a serious facial esthetic and dermatological problem among human^63^. On the contrary, the increased melanin synthesis and accumulation of these pigments occur in many types of skin disorders, including Acanthosis nigricans, Cervical Poikiloderma, melasma, Periorbital hyperpigmentation, Lentigines, neuro-degeneration associated with Parkinson’s disease and skin cancer risk^64–66^. Although melanogenesis is a complicated process represented by numerous enzymatic and chemical reactions, the enzymes such as tyrosinase and other tyrosinase-related proteins (TYRP1 and TYRP2) have a critical role in melanin synthesis. Tyrosinase is a multifunctional copper-containing metalloenzyme with dinuclear copper ions, which plays as a rate-limiting enzyme in the synthesis of melanin (Figure 1)^52^^,^^67^. Also, tyrosinase constitutes the primary cause for undesired browning of fruits and vegetables as well as diseases resulting from overproduction of melanin. Therefore, controlling the activity of enzyme by tyrosinase inhibitors is an essential endeavor for treating hypopigmentary disorders of mammals and enzymatic browning of fruits and fungi. To date, numerous effective inhibitors are identified and developed for using in the medical and cosmetic products, as well as food bioprocessing and agricultural industries and environmental industries. However, in medicine, tyrosinase inhibitors are a class of important clinical antimelanoma drugs but only a few compounds are known to serve as effective and safe tyrosinase inhibitors.

## Mushroom tyrosinase properties

Tyrosinases have been isolated and purified from different sources such as some plants, animals and microorganisms. Although many of them (such as human) have been sequenced, only few of them have been characterised. Recently, a novel tyrosinase produced by Sahara soil actinobacteria have been isolated and biochemically charactrised with the aim to identify novel enzymes with exclusive features for biotechnological applications^68–80^. However, among different sources of tyrosinase, mushroom tyrosinase from *Agaricus bisporus* is a major and cheap source of tyrosinase with high similarity and homology compared to human tyrosinase^78^. Because of these good properties, the structural, functional and biochemical characteristics of mushroom tyrosinase have been studied extensively as a model system for screening of tyrosinase inhibitors and melanogenic studies, enzyme-catalysed reactions and enzyme-inhibitor structural studies so far^78^^,^^8^,^1–90^. Tyrosinase from *Agaricus bisporus* is a 120 kDa tetramer with two different subunits, heavy and light^91^, which was the first isolated by Bourquelot and Bertrand^92^ in 1895. It has three domains and two copper binding sites which bind to six histidine residues and interact with molecular oxygen in the tyrosinase active site. Also, a disulfide linkage stabilise its structure^93^. Recently, a 50 kDa tyrosinase isoform from *Agaricus bisporus* (H-subunit) have been purified with a high specific tyrosinase activity of more than 38,000 U/mg^94^.

### Reaction mechanism

**Figure 2:**
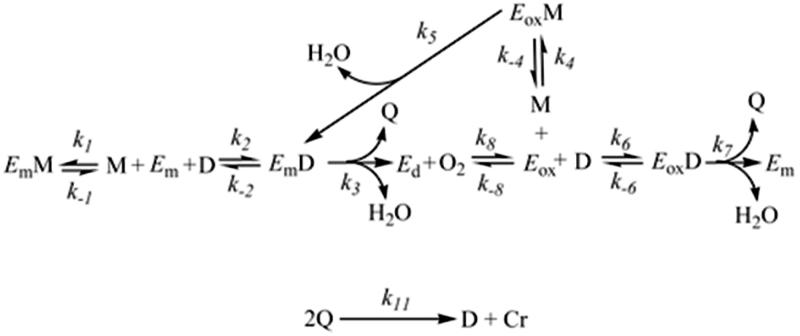
Monophenolase and diphenolase activities of Tyrosinase. E_m_M, met‐tyrosinase/monophenol complex; M, monophenol; D, *o-*diphenol; E_m_, met‐tyrosinase; E_m_D, met‐tyrosinase/*o*‐diphenol complex; E_d_, deoxy‐tyrosinase; O_2_, molecular oxygen; E_ox_, oxy‐tyrosinase; E_ox_D, oxy‐tyrosinase/*o*‐diphenol complex; E_ox_M, oxy‐tyrosinase/monophenol complex; Q, *o-*quinone; Cr, Dopachrome.

Tyrosinase (EC 1.14.18.1) has two activities in its catalytic cycle, see Figure 2^95^^,^^96^, a monophenolase activity where it hydroxylates monophenols (e.g l-tyrosine) to *o-*diphenols (e.g. l-dopa) and a diphenolase activity where tyrosinase oxidises *o-*diphenols to *o-*quinones (*o-*dopaquinone). At the same time of these enzymatic reactions, there are different chemical reactions coupled where two molecules of *o-*dopaquinone react their-selves generating an *o-*diphenol molecule (L-dopa) and a dopachrome molecule.

Diphenolase activity can be independently studied, when tyrosinase reacts with an *o-*diphenol (see Figure 2). The form met-tyrosinase (*E_m_*) binds the *o-*diphenol (D) originating the complex *E_m_*D. This complex oxidises the *o-*diphenols transforming it to *o-*quinone and the enzyme is converted into the form deoxy-tyrosinase (*E_d_*). *E_d_* has a very big affinity for the molecular oxygen originating the form oxy-tyrosinase (*E_ox_*), which binds another *o-*diphenol molecule and originating the complex *E_ox_*D. After that, the *o-*diphenol is oxidised again to *o*-quinone and the form E_m_ is formed again completing the catalytic cycle. However, after these enzymatic reactions, two *o-*quinone molecules (e.g. *o-*dopaquinone) react generating dopachrome and regenerating a molecule of *o-*diphenol.

As mentioned before, we can independently study the diphenolase activity. However, it is not applicable for the monophenolase activity, see Figure 2, because the chemical reactions of diphenolase activity have to occur at the same time of monophenolase activity. Tyrosinase shows the monophenolase activity with a lag period. This period is the time that the enzyme requires to accumulate a quantity of *o-*diphenol in reaction medium and is proportional to the quantity of monophenol used. Figure 2 shows the new complexes appeared in the monophenolase activity: *E_ox_*M (oxy-tyrosinase bound to monophenol) and *E_m_*M (met-tyrosinase bound to monophenols). *E_ox_*M is active and is transformed into *E_m_*D, which is an intermediate of the catalytic cycle^95^. *o*-Quinones formed by these two oxidation cycle spontaneously react with each other to form oligomers^97^.

## Tyrosinase inhibition

Due to the critical role of tyrosinase in the melanogenesis and browning process, several investigations have been reported for the identification of tyrosinase inhibitor from both natural (fungi, bacteria, plants) and synthetic sources so far. General speaking, tyrosinase inhibitors are examined in the presence of a monophenolic substrate such as tyrosine or a diphenolic substrate such as l-dopa, and activity is assessed based on dopachrome formation.

### Inhibition mechanism

**Figure 3:**
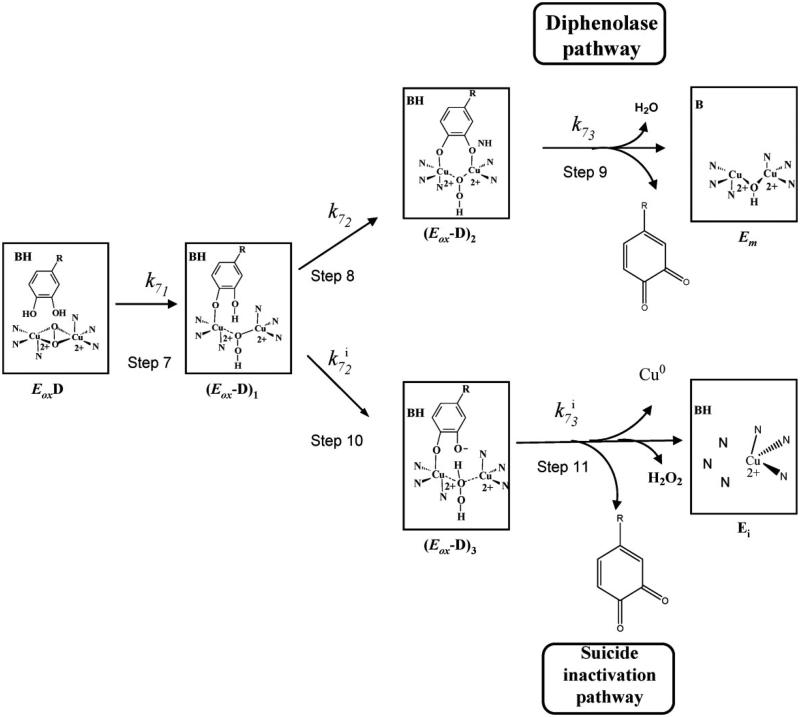
Detail of the structural mechanism proposed to explain the suicide inactivation of tyrosinase during its action on *o*‐diphenols. E_m_, met‐tyrosinase; E_ox_, oxy‐tyrosinase; E_ox_D, oxy‐tyrosinase/*o*‐diphenol complex; (E_ox_‐D)_1_, oxy‐tyrosinase/*o*‐diphenol complex axially bound to a Cu atom; (E_ox_‐D)_2_, oxy‐tyrosinase/*o*‐diphenol complex axially bound to the two Cu atoms; (E_ox_‐D)_3_, oxy‐tyrosinase/*o*‐diphenol complex axially bound to one Cu atom and the deprotonated hydroxyl group of C‐3; E*_i_*, inactive form of tyrosinase. A general view of this scheme is shown in Ref ^98^.

Among different types of compounds such as specific tyrosinase inactivators and inhibitors, *o*-dopaquinone scavengers, alternative enzyme substrates, nonspecific enzyme inactivators and denaturants, only specific tyrosinase inactivators and reversible inhibitors actually bind to the enzyme as true inhibitors and really inhibit its activity:

- Specific tyrosinase inactivators. They are called suicide inactivators or mechanism-based inhibitors. This group of compounds can be considered very interested from a pharmacological point of view, in hyperpigmentation processes (Figure 3)^98^.

To explain the suicide inactivation of tyrosinase, mainly two mechanisms have been proposed^98^^,^^99^. Accordingly, Haghbeen et al. have suggested that the conformational changes, triggered by the substrate then mediated by the solvent molecules, in the tertiary and quaternary structures of tyrosinase, might be the real reason for the suicide inactivation^100^. On the other hand, however, based on reports, it was found that acetylation of tyrosine residues with N-acetylimidazole protects mushroom tyrosinase from the suicide inactivation in the presence of its catecholic substrate, 4-[(4-methylbenzo) azo]-1,2-benzenediol without any major impact on the secondary structure of enzyme^101^.

The studies about the kinetics of suicide inactivation of tyrosinase have been carried out with several *o-*diphenolic substrates^102^, ascorbic acid^103^, l- and d-dopa^104^ and with different aminophenols and *o-*diamines^105^. The authors have established that the suicide inactivation could occur after the transference of a proton to the peroxide group on the active site of oxy-tyrosinase^98^^,^^106^, also it has been proposed that the monophenols do not inactivate the enzyme^107^^,^^108^.

The chemical structure of the different substrates is diverse, but the process always requires a step of oxidation/reduction: *o-*diphenols^102^^,^^104^, ascorbic acid^103^, aminophenols and *o-*diamines^105^, hydroxyhydroquinone^109^, tetrahydrobiopterines^110^, tetrahydrofolic acid^111^ and NADH^112^.

- Generally, the mode of inhibition by “true inhibitors” is one of these four types: competitive, uncompetitive, mixed type (competitive/uncompetitive), and noncompetitive. A competitive inhibitor can bind to a free enzyme and prevents substrate binding to the enzyme active site. Regarding the property that tyrosinase is a metalloenzyme, copper chelators such as many aromatic acids, phenolic and poly-phenolic compounds, a few non-aromatic compounds, can inhibit tyrosinase competitively by mimicking the substrate of tyrosinase^52^^,^^60^. Recently, it was found that d-tyrosine negatively regulates melanin synthesis by inhibiting tyrosinase activity, competitively^113^. In addition, l-tyrosine has been shown as an inhibitor^114^.

In contrast, an uncompetitive inhibitor can bind only to the enzyme-substrate complex and a mixed (competitive and uncompetitive mixed) inhibitor can bind to both forms of free enzyme and enzyme-substrate complex. Finally, noncompetitive inhibitors bind to a free enzyme and an enzyme–substrate complex with the same equilibrium constant^115^. Non-competitive and mixed-inhibition are frequent modes observed in the kinetics studies on mushroom tyrosinase activities. Phthalic acid and cinnamic acid hydroxypyridinone derivatives^116^ are two examples of mixed type inhibitors of mono-phenolase activity^117^. Also, some compounds such as phthalic acid^46^ and terephthalic acid^118^, D-(−)-arabinose^119^, brazilein^120^, thymol analogs^121^ were demonstrated as mixed-type effector examples of di-phenolase activity. Furthermore, other compounds such as bi-pyridine derivatives^122^, two thiadiazole derivatives^44^ barbarin^123^, chlorocinnamic acids^124^, propanoic acid^125^, some N-(mono- or dihydroxybenzyl)-N-nitrosohydroxylamines^126^ and *p-*alkylbenzaldehydes^127^ inhibited catecholase activity of mushroom tyrosinase uncompetitively. Some derivatives of thiazoles are examples for noncompetitive tyrosinase inhibition^128^.

In addition to determining the inhibition mechanism, inhibitory strength which is expressed as the IC_50_ value (the concentration of inhibitor at which 50% of your target is inhibited) should be calculated in the enzyme kinetics studies and inhibitor screening to compare the inhibitory strength of an inhibitor with others. However, the IC_50_ values may be incomparable due to the varied assay conditions (different substrate concentrations, incubation time, and different sources of tyrosinase) but a positive control can be used for this purpose^52^. Although, some researchers have not calculated IC_50_ and have not applied a positive control in their studies but, fortunately, in most studies conducted for screening new tyrosinase inhibitors, the popular whitening agents, such as kojic acid, arbutin or hydroquinone, were used as a positive control^129^ at the same time. However, among different types of mushroom tyrosinase inhibitors, some inhibitors such as hydroquinone^49^ arbutin, kojic acid^15^^,^^49^ , azelaic acid, l-ascorbic acid, ellagic acid and tranexamic acid have been reported as skin-whitening agents in the cosmetic industry but there are a few reports failed to confirm their effect as an agent to lighten skin in clinical trials despite the safety of this compound^5^. Recently, Mann et al., have compared the inhibitory effects of hydroquinone, arbutin and kojic acid by human tyrosinase and mushroom tyrosinase. They have found hydroquinone and arbutin and kojic acid (IC_50_ > 500 µmol/L) weekly inhibits human tyrosinase. In contrast, a resorcinyl-thiazole derivative, thiamidol, is a most potent inhibitor of human tyrosinase (IC_50_ of 1.1 µmol/L) but inhibits mushroom tyrosinase weakly (IC_50_ = 108 µmol/L)^130^. Also, deoxyarbutin, a novel reversible tyrosinase inhibitor with effective *in vivo* skin lightening potency, have been reported due to its increased skin penetration and binding affinity to human tyrosinase^131^. In another research, Sugimoto et al. have investigated a comparison of inhibitory effects of alpha-arbutin and arbutin with human tyrosinase and they have found α-arbutin is stronger than arbutin^132^.

## Natural tyrosinase inhibitor sources

Natural sources including plants, bacteria and fungi have recently become of increasing interest for their antityrosinase activity by producing bioactive compounds. A number of researchers prefer to identify inhibitors from natural sources due to their less toxicity and better bioavalibility, especially for food, cosmetic and medicinal applications.

### Plants

It is well known that phenolic compounds are the largest group of phytochemicals found in plants, which are mainly the factors responsible for the activities in plant extracts^52^. Tyrosinase inhibitory activity of many plant extracts was carried out to find new sources of anti-tyrosinase compounds. For example, anti-tyrosinase activities of the following plants have been reported by various researchers: *Asphodelus microcarpus*^133^, *Morus nigra* L^134^, *Greyia radlkoferi Szyszyl*^45^, *Limonium tetragonum*^135^, *Arctostaphylos uva-ursi*^136^, *Pleurotus ferulae*^137^, *Agastache rugosa* Kuntze fermented with *Lactobacillus rhamnosus* and *Lactobacillus paracasei*^138^, *Artemisia aucheri* Boiss^139^, *Cassia tora*^140^, *S. brevibracteata* subsp^141^, *Rhodiola crenulata, Alpinia officinarum* Hance *and Zanthoxylum bungeanum* Maxim^142^, *Mangifera indica*^143^, *Podocarpus falcatus*^144^, *Momordica charantia*^142^, *Cymbopogon citrates*^145^, *Greyia flanaganii* (IC_50_ = 32.62 µg/ml)^146^, *Vitis vinifera* Leaf extracts (IC_50_ = 3.84 mg/mL)^147^ and *Inula britannica* L.^146^. Also, tyrosinase inhibitory activity of 91 native plants from central Argentina was carried out by Chiari et al.^138^^,^^147^. Their results approved the inhibitory activity of these extracts against tyrosinase: *Achyrocline satureioides, Artemisia verlotiorum, Cotoneaster glaucophylla, Dalea elegans, Flourensia campestris, Jodina rhombifolia, Kageneckia lanceolata, Lepechinia floribunda, Lepe-chinia meyenii, Lithrea molleoides, Porlieria microphylla, Pterocaulon alopecuroides, Ruprechtia apetala, Senna aphylla, Sida rhombifolia, Solanum argentinum, Tagetes minuta, and Thalictrum decipiens.* Besides, plants from the *Moraceae* family including genera *Morus* species, *Artocarpus*, *Maclura (Cudrania)*, *Broussonetia*, *Milicia (Chlorophora)*, and *Ficus* have shown *in vitro* tyrosinase inhibition^148^. Also, ethanolic and methanolic extracts of some other plants such as *Ardisia elliptica* Thunb^149^, *Phyllanthus acidus* (L.) Skeels, *Rhinacanthus nasutus* L. Kurz (IC_50_ value of 271.50 µg/ml), *Arbutus andrachne* L. (IC_50_ = 1 mg/mL)^150^, *Withania somnifera* L. Dunal and *Solanum nigrum* L. berries^151^, *Pulmonaria officinalis* and *Centarium umbellatum*^152^ and Camel’s foot creeper leaves (*Bauhinia vahlii*)^153^ significantly inhibited tyrosinase activity, too. Quispe et al. have screened tyrosinase inhibitory properties of Peruvian medicinal plants. Among these plant extracts, *Hypericum laricifolium* Juss*, Taraxacum officinale* F.H.Wigg. (IC_50_ value of 290.4 µg/ml), and *Muehlenbeckia vulcanica* Meisn (IC_50_ value of 280.1 µg/ml) showed the greatest anti-tyrosinase activity^154^. Furthermore, tyrosinase inhibitory activity of mangrove plants in Micronesia^155^, Korean indigenous plants^156^, plants from Brazilian Cerrado^157^, five traditional medicinal plants from Iran^158^, ethanol extracts from medicinal and edible plants cultivated in Okinawa^159^, seashore plants^160^, some tropical plants^161^ and Bangladeshi indigenous medicinal plants^162^, have been investigated by various researchers. Bonesi et al. have reported recent trends in the discovery of tyrosinase inhibitors from plant sources^163^.

### Fungi and bacteria

Fungi from different genera such as *Aspergillus* sp.^164^, *Trichoderma* sp.^165^, *Paecilomyces* sp.^166^, *Phellinus linteus*^167^, *Daedalea dickinsii*^168^, *Dictyophora indusiata*^169^ along with a liquid culture of *Neolentinus lepideus*^170^ have been reported as a source of novel tyrosinase inhibitor by producing bioactive compounds. Also, there have been several reports on tyrosinase inhibitors from some marine fungi species such as *Myrothecium* sp. isolated from algae^171^ and *Pestalotiopsis* sp. Z233^172^. Also, there are several reports on tyrosinase inhibition by bacterial species and their metabolites. Among them, *Streptomyces* sp., such as *S. hiroshimensis* TI-C3 isolated from soil^173^, an actinobacterium named *Streptomyces swartbergensis* sp. Nov.^174^ and *Streptomyces roseolilacinus* NBRC 12815^175^ are potential bacterial sources of tyrosine inhibitors. Moreover, some tyrosinase inhibitors have been reported from a gram-negative marine bacterium *Thalassotalea* sp. Pp2-459^176^ and a toxic strain of the cyanobacterium, *Oscillatoria agardhii*^177^. Interestingly, some probiotics such as *Lactobacillus* sp.^178^ which are used in the fermentation process have been investigated as natural tyrosinase inhibitor sources. Based on the studies, it has been confirmed that the physiological activities of fermented extracts are considerably higher than those of unfermented extracts and their cytotoxic activity is lower as compared to unfermented extracts^179^. Recently, tyrosinase inhibitory four different lactic acid bacteria (LAB) strains isolated from dairy cow feces have been proved by Ji et al.^180^.

Finally, in an updated review by Fernandes from reported findings, tyrosinase inhibitors produced by microorganisms have been summarised^61^. This review shows that diverse tyrosinase inhibitors isolated from plant sources and fungi are mostly phenolic compounds, steroids, and alkaloids structurally comparable with each other. In contrast, tyrosinase inhibitors from bacteria comprise a smaller group of alkaloids, macrolides, and polyphenols, which competitively inhibit the enzyme^61^.

## Inhibitors from natural, semisynthetic and synthetic sources

### Simple phenols

Phenolic compounds which are characterised by having at least one aromatic ring and one (or more) hydroxyl group can be classified based on the number and arrangement of their carbon atoms. These compounds are commonly found to be conjugated to sugars and organic acids. Phenolics range from simple to large and complex tannins and derived polyphenols due to their molecular-weight and number of aromatic-rings^180^.

**Figure 4:**
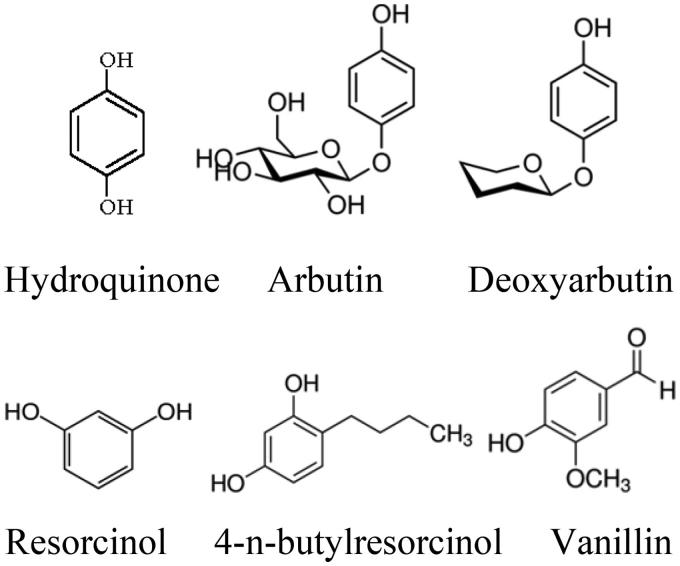
Chemical structures of some simple phenolic compounds.

The simple phenols such as hydroquinone^181^^,^^182^ and its derivatives^183^^,^^184^, deoxyarbutin^185^^,^^186^ and its derivatives^187^, 4–(6-Hydroxy-2-naphthyl)-1,3-bezendiol, resorcinol (or resorcin)^188^ and 4-n-butylresorcinol^189^, vanillin^190^ and its derivatives^191^^,^^192^ have been reported in the scientific literature as possible phenolic inhibitors of the tyrosinase (Figure 4). Chen et al. have found the alkylhydroquinone 10'(Z)-heptadecenylhydroquinone, isolated from the sap of the lacquer tree *Rhus succedanea*, can inhibit the activity of tyrosinase and suppress melanin production in animal cells. The IC_50_ of this compound (37 µM) is less than hydroquinone (70 µM) as a known inhibitor of tyrosinase. They have suggested that the potent inhibitory effect of this derivative on tyrosinase activity is likely due to its heptadecenyl chain, which facilitates the oxidation of the hydroquinone ring^183^^,^^184^.

Isotachioside, a methoxy-hydroquinone-1-O-beta-d-glucopyranoside isolated from *Isotachis japonica* and *Protea neriifolia* and its glycoside derivatives (glucoside, xyloside, cellobioside, and maltoside) are categorised as analogs of arbutin. However, isotachioside and arbutin could not be determined as potent inhibitor. But, glucoside, xyloside, cellobioside and maltoside derivatives, missing methyl and benzoyl groups, acted as tyrosinase inhibitors with IC_50_s of 417, 852, 623 and 657 µM, respectively. Among these novel inhibitors, glucoside derivative (IC_50_ = 417 µM) was the most potent, indicating that the structural combination of resorcinol and glucose was significant for inducing the inhibitory effect^193^.

Hydroquinone and some of its known derivatives, including α and β-arbutin, are described as both a tyrosinase inhibitor and a substrate^194^^,^^195^. Deoxyarbutin and its second-generation derivatives have been proposed as promising agents to ameliorate hyperpigmented lesions or lighten skin due to less toxicity at their effective inhibitory dose^185^^,^^186^.

Monophenolic compounds such as l-tyrosine, l-α-methyl-tyrosine and tyramine are substrates of tyrosinase. *o-*Quinone evolves in the medium of reaction accumulating *o-*diphenol and this accumulation provokes that met-tyrosinase (E_m_) is transformed into oxy-tyrosinase (E_ox_), which is the active form of the tyrosinase for monophenols and *o-*diphenols. Therefore, tyrosinase is active with monophenols such as: umbelliferone^196^, hydroquinone^197^^,^^198^*p-*hydroxybenzyl alcohol^199^, 4-hexylresorcinol^200^, oxyresveratrol^201^, 4-*n*-butylresorcinol^202^, resorcinols^203^, α and β-arbutin^195^ and *p-*coumaric acid^204^^,^^205^ when we add the following reagents to medium of reaction: hydrogen peroxide (transforms *E_m_* to E_ox_), an *o-*diphenol or a reducing agent such as ascorbic acid transforming E_m_ to E_d_ which, with molecular oxygen, is transformed into E_ox_. A particular case is deoxyarbutin, which acts as a substrate of tyrosinase even if any reagent is not added to the medium of reaction^206^. Taking into consideration all the previous comments, several methods have been developed to discriminate between true inhibitors and alternative substrates of the enzyme^98^^,^^207^.

### Polyphenols

Plants produce a large diverse class of polyphenols including phenolic acids, flavonoids, stillbenes and lignans^208^^,^^209^. A large number of these compounds have been reported as a weak or potent inhibitor of tyrosinase from natural^210–215^ and synthetic^216–219^ sources.

#### Flavonoids

Among polyphenolic compounds, some of the flavonoid derivatives mostly found in herbal plants, fruits and synthetic sources have been raveled to be the potent inhibitors of tyrosinase^133^^,^^211^^,^^220^,^225^. There is a significant correlation between the inhibitory potency of flavonoids on mushroom tyrosinase and melanin synthesis in melanocytes^226^. In searching effective tyrosinase inhibitors from natural products, many flavonoid compounds have been isolated and evaluated for their inhibitory activity on mushroom tyrosinase from different natural sources such as *Trifolium nigrescens* Subsp*. Petrisavi*^227^, mung bean (*Vigna radiatae* L.)^228^, *calamondin* peel^229^, *Morus yunnanensis*^230^, *Bhagwa* and *Arakta cultivar*^231^, *Tibouchina semidecandra* L^232^, *Maackia faurie*^232^, *Pleurotus ostreatus*^233^, *Potentilla bifurca*^234^, *Alpinia officinarum*^235^, roots of *Morus lhou*^236^, *Garcinia subelliptica*^160^, *Artocapus altilis*^190^, *Myrsine africana*^237^, *Pulsatilla cernua*^238^, *Salvia miltiorrhiza*-*Carthamus tinctorius* (Danshen-Honghua, DH) herbal pair^239^ and other various medicinal plants^240^.

**Figure 5:**
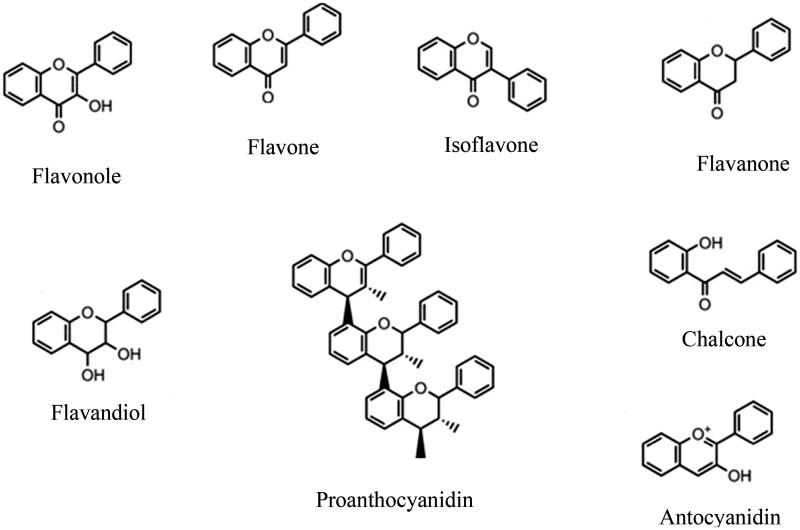
Structure of the main classes of flavonoids.

Generally, major flavonoids (Figure 5) are classified into several main classes: flavones, flavonols, isoflavones, flavanones, flavanoles and anthocyanidins. Minor flavonoids included: dihydroflavones, flavan-3,4-diols, coumarins, chalcones, dihydrochalcones and aurones^241^. Also, prenylated and vinylated flavonoids, such as flavonoid Glycosides, are other subclasses of flavonoids. Some flavonoid glycosides such as myricetin 3-galactoside and quercetin 3-O-β-galactopyronaside from *Limonium tetragonum*^133^ and 3',5'-di-C-β glucopyranosylphloretin from unripe calamondin peel (IC_50_ = 0.87 mg/ml)^229^, have been investigated for their inhibitory activities on tyrosinase. Moreover, the inhibitory activities of some other prenylated and vinylated flavonoids, such as kuwanon C, papyriflavonol A, sanggenon D and sophoflavescenol, and sanggenon D (IC_50_ = 7.3 µM) against tyrosinase, have been approved by Lee et al.^242^. However, according to their findings, the prenylation with isoprenyl group or the vinylation of some flavonoid molecules does not enhance their tyrosinase inhibitory activity^242^. Interestingly, it has even demonstrated that deglycosylation of some flavonoid glycosides by far-infrared irradiation can be improved tyrosinase inhibitory activity^243^. In a survey from reported findings (2008–2013), Orhan et al. reviewed many examples of tyrosinase inhibitors with flavonoid structure^220^. In the following, some tyrosinase inhibitors from various flavonoid classes have been mentioned and discussed.

##### Flavones and dihydroflavones

The most common flavones are luteolin, apigenin, baicalein, chrysin and their glycosides (e.g. apigetrin, vitexin, and baicalin)^209^. Furthermore, nobiletin and tangeretin are the polymethoxylated flavones^244^. Nguyen et al. have investigated the presence of apigenin and nobiletin from the methanolic extract of the heartwood of *Artocapus altilis* with 11 other phenolic compounds for their inhibitory activities on tyrosinase^190^. In another research, Shang et al. have found a derivative of flavone, namely 7,8,4´-trihydroxyflavone which inhibits diphenolase activity of tyrosinase with an IC_50_ value of 10.31 ± 0.41 µM and a noncompetitive manner with a *K_i_* of 9.50 ± 0.40 µM. The quenching anlaysis of tyrosinase by this compound showed a static mechanism and a single binding site with a binding constant of 7.50 ± 1.20 × 10^4^  M^−1^ at 298 K. Based on the thermodynamics parameters, the binding process involved hydrogen bonds and van der Waals forces. Also, docking simulation illustrated hydrogen bonds between this compound and the residues His244 and Met280 of active site^245^.

In addition, several hydroxyflavones including baicalein, 6-hydroxyapigenin, 6-hydroxygalangin and 6-hydroxy-kaempferol^246^ and tricin (5,7,4′-trihydroxy-3′,5′-dimethoxyflavone)^247^ have been demonstrated as inhibitors of diphenolase activity of tyrosinase. The mechanism of inhibition by baicalein (IC_50_ = 0.11 mM) indicated a mix-type (*K_i_* of 0.17 mM, *α* = 0.56). A single binding site with a binding constant of 2.78 × 10^5^ M ^− 1^ was obtained from the quenching fluorescence analysis for this compound. Thermodynamic parameters suggested spontaneous binding through hydrogen bonding and van der Waals forces. Furthermore, circular dichroism spectra indicated a reduction in the content of α-helix from 32.67% to 29.00% due to this binding. Docking simulations also indicated that baicalein mainly bound tyrosinase via its Met280 residue^248^. While, tricin was found as a noncompetitive inhibitor of tyrosinase with good efficacy compared to its control. Based on circular dichroism spectra, the interactions between tricin and tyrosinase did not change the secondary structure. Fluorescence quenching revealed that the interaction of tricin with residues in the hydrophobic pocket of tyrosinase is stabilised by hydrophobic interactions and hydrogen bonding. Also, docking results implied that the stereospecific effects of tricin on substrates or products and flexible conformation alterations of tyrosinase produced by weak interactions between tricin and this enzyme are the possible inhibitory mechanisms of this compound^247^.

Another flavone named morusone from the twigs of *Morus alba* L. (IC_50_ = 290.00 ± 7.90 µM)^249^, a new bioflavone 4''',5,5″,7,7″-pentahydroxy-3',3'''-dimethoxy-3-O-β-d-glucosyl-3″,4'-O-biflavone from *Trifolium nigrescens* Subsp. Petrisavi^227^, along with apigenin, flavone glucoside vitexin (IC_50_ = 6.3 mg/ml) and a C-glycosylflavone isovitexin (IC_50_ = 5.6 mg/ml) from *Vigna radiatae L*. extracts exhibited significant tyrosinase inhibition activities^228^. Also, inhibitory effects of five flavones including mormin (IC_50_ = 0.088 mM), cyclomorusin (IC_50_ = 0.092 mM), morusin (IC_50_ = 0.250 mM), kuwanon C (IC_50_ = 0.135 mM) and norartocarpetin (IC_50_ = 1.2 µM) isolated from the stem barks of *Morus lhou* (S.) Koidz, have been investigated by Ryu et al. The mechanism of inhibition indicated that mormin, cyclomorusin, kuwanon C and norartocarpetin inhibited tyrosinase competitively^250^.

##### Flavonoles

Myricetin, kaempferol, quercetin, morin, isorhamnetin, galangin and their glycosides (e.g. rutin, quercitrin, and astragalin) are the predominant flavonols most commonly found as *O*-glycosides^209^. So far, several flavonols such as kaempferol from *Hypericum laricifolium* Juss^154^ and *Crocus sativus* L.^251^, quercetin from *Olea europaea* L.^252^, quercetin-4'-O-beta-d-glucoside from *Potentilla bifurca*^253^, quercetin-3-O-(6-O-malonyl)-β-d-glucopyranoside and kaempferol-3-O-(6-O-malonyl)-β-d-glucopyranoside from mulberry leaves^253^, galangin from *Alpinia officinarum*^235^, morin^254^ and (±) 2,3-cis-dihydromorin (IC_50_ = 31.1 µM), 2,3-trans-dihydromorin (IC_50_ = 21.1 µM) from *Cudrania cochinchinensis*^255^, were identified as tyrosinase inhibitors.

Based on kinetics studies, morin reversibly inhibited tyrosinase through a multi-phase kinetic process and bind to tyrosinase at a single binding site mainly by hydrogen bonds and van der Waals forces. It inhibited tyrosinase reversibly in a competitive manner with *K_i_* = 4.03 ± 0.26 mM and the binding of morin to tyrosinase-induced rearrangement and conformational changes of the enzyme^254^. Furthermore, it was reported that three flavonols including galangin^235^, kaempferol^251^ and quercetin inhibit the oxidation of L-DOPA catalysed by mushroom tyrosinase and presumably this inhibitory activity comes from their copper chelating ability. While their corresponding flavones, chrysin, apigenin and luteolin, are not identified as copper chelator, Kubo et al. believed that the chelation mechanism by flavonols may be attributed to the free 3-hydroxyl group^251^. Interestingly, quercetin behaves as a cofactor and does not inhibit monophenolase activity. In contrast, galangin inhibits monophenolase activity and does not act as a cofactor, and kaempferol neither acts as a cofactor nor inhibits monophenolase activity. However, inhibiting of diphenolase activity by chelating copper in the enzyme is the common feature of these three flavonols^160^.

Recently, 8-prenylkaempferol as a competitive tyrosinase inhibitor along with Kushenol A (noncompetitive) isolated from *Sophora flavescens*^256^, have been investigated with IC_50_ values less than 10 µM. Finally, based on the literature review, many flavonol inhibitors are usually competitive inhibitors due to the 3-hydroxy-4-keto moiety of the flavonol structure, which chelates the copper in the active site ^251^. Also, among all these compounds, quercetin-4'-O-beta-d-glucoside with a IC_50_ value of 1.9 µM is revealed stronger tyrosinase inhibition than their positive control, kojic acid^236^. While the other flavonol inhibitors listed above are very weak inhibitors and have little potential as skin whitening or food antibrowning.

##### Isoflavones

Isoflavones such as daidzein, genistein, glycitein, formononetin, and their glycosides (e.g. genistin, daidzin) mostly are detected in the medicinal herbs^209^. Park et al. have investigated tyrosinase inhibition activities of some natural o-dihydroxyisoflavone derivatives with variable hydroxyl substituent at the aromatic ring of isoflavone isolated from five-year-old Korean fermented soybean paste. They have demonstrated that two derivatives 7,8,4'-trihydroxyisoflavone and 7,3',4'-trihydroxyisoflavone inhibit tyrosinase by IC_50_ value of 11.21 ± 0.8 µM and 5.23 ± 0.6 µM, respectively, whereas very low inhibition activity was obtained for 6,7,4'-trihydroxyisoflavone, daidzein, glycitein and genistein^257^. Also, 6,7,4'-trihydroxyisoflavone was identified as a potent competitive inhibitor of monophenolase activity of tyrosinase by Chang et al., with an IC_50_ value of 9.2 µM, which is six times potent than kojic acid^258^. But, its analogs, glycitein, daidzein, and genistein showed little anti-tyrosinase activity. Therefore, they have suggested that C-6 and C-7 hydroxyl groups of the isoflavone skeleton might play an important role in the tyrosinase inhibitory activity. Furthermore, two other isoflavone metabolites, 7,8,4'-trihydroxyisoflavone and 5,7,8,4'-tetrahydroxyisoflavone isolated from soygerm koji, were investigated by Chang et al.^259^. These compounds inhibited both monophenolase and diphenolase activities with an irreversible inhibition manner. Interestingly, by using HPLC analysis and kinetic studies, they have found that 7,8,4'-trihydroxyisoflavone and 5,7,8,4'-tetrahydroxyisoflavone are potent suicide substrates of mushroom tyrosinase. It may be concluded that the hydroxyl groups at both the C7 and C8 positions could completely change the inhibitory mechanism of the isoflavones from the reversible competitive to the irreversible suicide form^52^.

Recently, a noncompetitive inhibitor, glabridin (IC_50_ = 0.43 µM), isolated from the root of *Glycyrrhiza glabra* Linn, has exhibited excellent inhibitory effects on tyrosinase. The quenching analysis of tyrosinase by glabridin showed a static mechanism^260^. Notably, a drug delivery system by using glabridin microsponge-loaded gel as a new approach for hyperpigmentation disorders have been proposed by Deshmukh et al.^261^. In another research, Jirawattanapong et al. have identified a synthetic glabridin, 3'',4''-dihydroglabridin, with higher activity than glabridin (IC_50_ = 11.40 µM) against tyrosinase. They have suggested the more effective interaction with the enzyme may be due to more conformational flexibly of this compound that has occurred by the 4-substituted resorcinol skeleton and the lacking of double bond between carbon atom 3'' and 4'' in its structure^262^. Also, Nerya et al. have reported that another isoflavone, glabrene, in the licorice extract can inhibit both monophenolase and diphenolase tyrosinase activities^263^. In the study reported by Heo et al., two new isoflavones desmodianone H and uncinanone B have been identified as novel tyrosinase inhibitors. However, uncinanone B has higher anti-tyrosinase rate than desmodianone H^264^. Glyasperin C from *Glycyrrhiza glabra* is another kind of isoflavone identified as tyrosinase inhibitor^265^. Furthermore, some other isoflavones, formononetin, genistein, daidzein, texasin, tectorigenin, odoratin and mirkoin isolated from the stems of *Maackia fauriei*, have been investigated by Kim et al. for their tyrosinase inhibition activity. Based on their results, among these falvonoids, mirkoin (IC_50_ = 5 µM) revealed stronger tyrosinase inhibition than the positive control, kojic acid and inhibited tyrosinase reversibly in a competitive mode^232^. Recently, two isoflavonoids lupinalbin (IC_50_ = 39.7 ± 1.5 µM), and 2′-hydroxygenistein-7-O-gentibioside (IC_50_ = 50.0 ± 3.7 µM) from *Apios americana* were identified as competitive inhibitors, with *K_i_* values of 10.3 ± 0.8 µM and 44.2 ± 1.7 µM, respectively^266^.

##### Flavanones

Flavanones such as naringenin, hesperetin, eriodictyol and their glycosides (e.g. naringin, hesperidin, and liquiritin) and flavanonols (taxifolin) are mainly found in citrus fruits and the medicinal herbs^209^. A copper chelator flavanone named hesperetin inhibits tyrosinase reversibly and competitively. Based on the ANS-binding fluorescence analysis, hesperetin disrupted of tyrosinase structure by hydrophobic interactions. In addition, hesperetin chelates a copper ion coordinating with 3 histidine residues (HIS61, HIS85, and HIS259) within the active site pocket of the enzyme due to docking simulation results^267^. In another study, Chiari et al. have illustrated tyrosinase inhibitory activity of a 6-isoprenoid-substituted flavanone isolated from *Dalea elegans*^268^. Also, Steppogenin is a natural flavanone with a strong tyrosinase inhibitory activity (IC_50_ = 0.98 ± 0.01 µM), from *Morus alba* L^249^. Recently, a new isoprenylated sanggenon-type flavanone, nigrasin K, along with some other analogs including sanggenon M, C and O, chalcomoracin, sorocein H and kuwanon J isolated from the twigs of *Morus nigra* have been identified as potent tyrosinase inhibitors by Hu et al.^269^. Among these natural inhibitors, sanggenon D revealed stronger tyrosinase inhibition than the positive control, kojic acid or arbutin.

##### Flavanoles and flavan-3,4-diols

Flavan-3-ols are the most complex subclass of flavonoids ranging from the simple monomers (+)-catechin and its isomer (−)-epicatechin to the oligomeric and polymeric proanthocyanidins, which are also known as condensed tannins. Flavanols, such as catechin, epicatechin, epi-gallocatechin, epicatechin gallate (ECG), epigallocatechin gallate (EGCG) and proanthocyanidins are widespread in the medicinal herbs and higher plants^231^^,^^270^. *Alphitonia neocaledonica* (Rhamnaceae) is an endemic tree of New Caledonia, which has been identified as an anti-tyrosinase source due to the presence of tannins and gallocatechin^228^. Moreover, a catechin compound isolated from the ethanol extract of *Distylium racemosum* branches, with IC_50_ value of 30.2 µg/mL, showed higher tyrosinase inhibition activity than arbutin as a positive control^271^. Also, a proanthocyanidins from *Clausena lansium* demonstrated potent mushroom tyrosinase inhibition in a mixed competitive manner and illustrated strong inhibition of the melanogenic activity of B16 cells. The IC_50_ values for the monophenolase and diphenolase activities were 23.6 ± 1.2 and 7.0 ± 0.2 µg/mL, respectively. Furthermore, from the inhibition mechanism of this compound, it can be concluded that a chelation between the hydroxyl group on the B ring of the proanthocyanidins and dicopper ions of the enzyme has been occurred^39^.

Another investigation revealed that procyanidin-type proanthocyanidins, purified from cherimoya (*Annona squamosa)* pericarp could powerfully inhibit the activities of monophenolase and diphenolase of tyrosinase, competitively^272^. In addition, Kim et al. have demonstrated that (+)-catechin-aldehyde polycondensates inhibit the l-tyrosine hydroxylation and L-DOPA oxidation by chelation to the active site of tyrosinase^273^. Recently, another tyrosinase inhibitor from this class, condensed tannins (mixtures of procyanidins, propelargonidins, prodelphinidins) and their acyl derivatives (galloyl and p-hydroxybenzoate) from *Longan Bark* indicated the reversible and mixed (competitive is dominant) inhibition of tyrosinase^274^.

##### Anthocyanidins

Anthocyanins, including anthocyanidins (e.g. cyanidin, delphinidin, malvidin, peonidin, pelargonidin, etc.) and their glycosides, are widely distributed in the medicinal herbs^217^. It seems that there is a significant relationship between anthocyanin content with anti-human and anti-mushroom tyrosinase activities^275^.

##### Curcuminoids

Two phenolic compounds, namely curcumin and desmethoxycurcumin have been isolated from the methanolic extract of the heartwood of *Artocapus altilis* and showed more potent tyrosinase inhibitory activities than the positive control kojic acid^190^. Also, a curcumin included in Chouji and Yakuchi extracts inhibited the enzyme competitively^192^. In addition, some synthetic curcumin derivative compounds^217^^,^^276^ and its analogs possessing *m-*diphenols and *o-*diphenols have been investigated as potent inhibitors of mushroom tyrosinase^216^. Based on the results, 4-hydroxyl groups in curcumin analogs containing 4-hydroxyl-substituted phenolic rings with C-2/C-4- or C-3/C-4-dihydroxyl-substituted diphenolic rings make them more active than kojic acid^217^.

##### Coumarins

**Figure 6:**
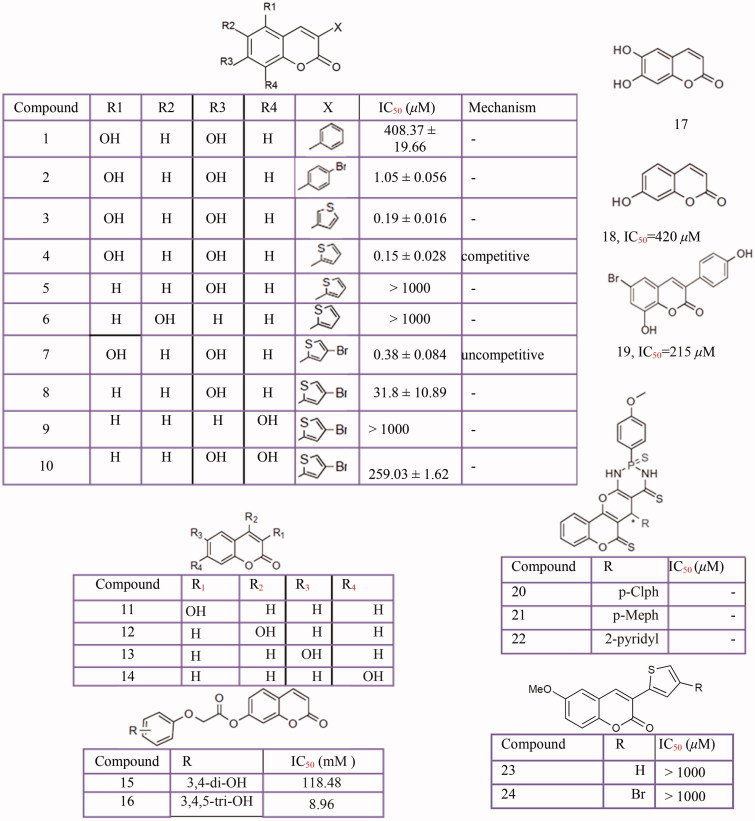
Inhibitory effects of the coumarins derivatives against mushroom tyrosinase activity: 3-aryl and 3-heteroarylcoumarins **(1–10, 23–24)**, 3-hydroxycoumarin **(11)**, 4-hydroxycoumarin **(12)**, 6-hydroxycoumarin **(13)**, 7-hydroxycoumarin **(14)**, umbelliferone analogs **(15–16)**, Esculetin **(17)** umbelliferone **(18)**, 3-phenyl coumarins with bromo substituent **(19)**, thiophosphonic acid diamides **(20–22)**.

In search of tyrosinase inhibitors, the inhibitory effects of several coumarin derivatives (Figure 6)^277–279^ such as 3-aryl and 3-heteroarylcoumarins^280^, esculetin^281^, coumarinolignoid 8'-epi-cleomiscosin^282^, umbelliferone and their analogs^283^, phenyl coumarins^284^, hydroxycoumarins^285^^,^^286^, thiophosphonic acid diamides, diazaphosphinanes coumarin derivatives^287^, cardol-coumarin derivatives^288^ and coumarin-resveratrol hybrids^289^, were evaluated on tyrosinase activity.

Interestingly, among hydroxycoumarins, the 3-hydroxycoumarin^286^ and 7-hydroxycoumarin showed potent activity for the tyrosinase inhibition^278^, while the 4-hydroxycoumarin is not an inhibitor^286^. Also, 2-(1-(coumarin-3-yl)-ethylidene) hydrazinecarbothioamide and 2-(1-(6-chlorocoumarin-3-yl) ethylidene)-hydrazinecarbothioamide demonstrated an irreversible inhibition of tyrosinase^277^. Recently, in the screening of natural products for the development of cosmetic ingredients, two major compounds, *trans*-N-coumaroyltyramine (IC_50_ = 40.6 µM) and *cis*-N-coumaroyltyramine (IC_50_ = 36.4 µM) from *Humulus japonicus* showed potent tyrosinase inhibition^290^.

##### Chalcones and dihydrochalcones

**Figure 7:**
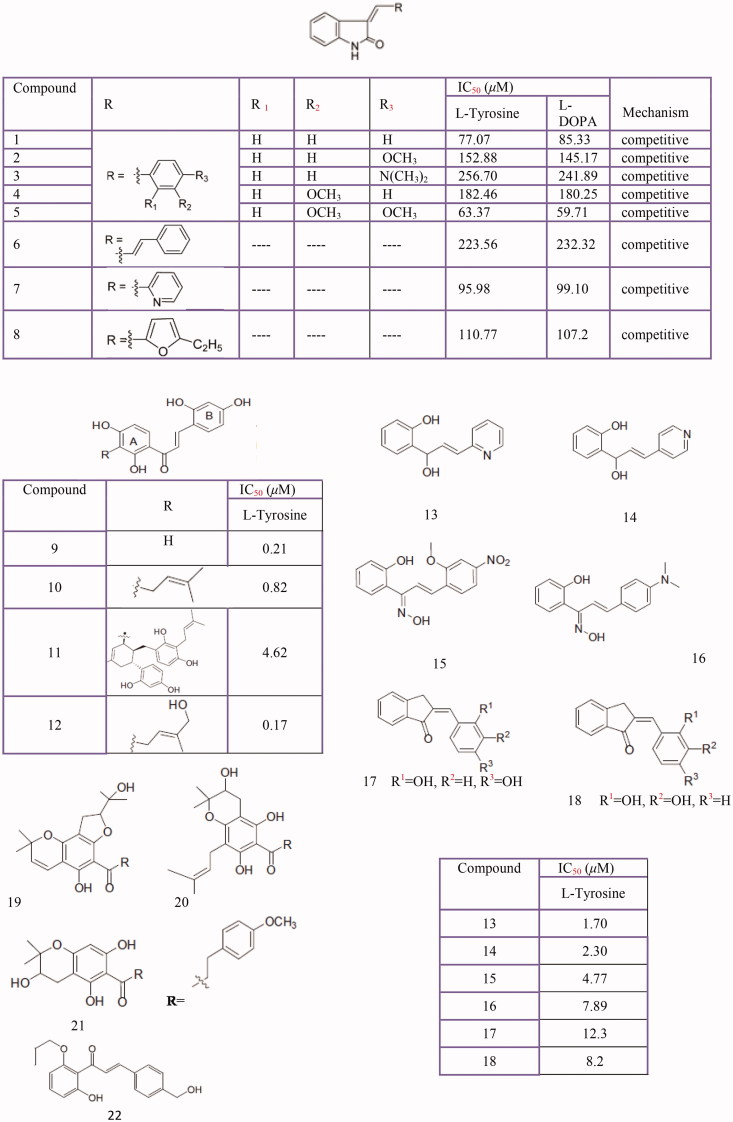
Tyrosinase Inhibition Activity of chalcone derivatives inhibitors: Oxindole-based chalcone **(1–8)**, chalcones isolated from *Morus australis***(9–12)** azachalcones **(13–14)**, oxime based chalcone series **(15,16)** 2,3-dihydro-1H-inden-1-one chalcone-like derivatives **(17,18)**, Dihydrochalcones from *Flemingia philippinensis***(19–21).** chalcone **(22)**.

Chalcones (butein, phloretin, sappan-chalcone, carthamin, etc.), or 1,3-diphenyl-2-propen-1-ones, are one of the most important classes of flavonoids. Chalcone-containing plants have been used for a long time in traditional medicine^209^. Based on the reports, some natural and synthetic chalcones and their derivatives are identified as new potent depigmentation agents and tyrosinase inhibitors (Figure 7). So far, natural chalcones isoliquiritigenin (2′,4′,4-trihydroxychalcone) and glabrene from licorice roots^283^, 2,4,2',4'-hydroxycalcone and three of its analogs with 3'-substituted resorcinol moieties from *Morus australis* (Figure 6, **19**–**22**)^291^, 2,4,2',4'-tetrahydroxy-3-(3-methyl-2-butenyl)-chalcone from *Morus nigra*^292^, vulpinoideol B from *Carex vulpinoidea* seeds^293^, dihydrochalcones from *Flemingia philippinensis*^210^, 2,4,2′,4′-tetrahydroxychalcone (IC_50_ = 0.07 ± 0.02 µM) and morachalcone A (IC_50_ = 0.08 ± 0.02 µM) from *Morus alba* L.^249^ and bavachinin from *Psoralea corylifolia*^21^ have been presented as tyrosinase inhibitors.

Also, tyrosinase inhibitory effects of several synthetic chalcones and their derivatives were evaluated by various researchers. Oxindole-based chalcones^294^, 1-(2-cyclohexylmethoxy-6-hydroxy-phenyl)-3-(4-hydroxymethyl-phenyl) propenone derivative^295^, isoxazole chalcone derivatives^296^, some azachalcones and their oximes^297^^,^^298^, 2,4,2',4'-tetrahydroxychalcone and its two derivatives (1,3,5-tris-(2,4-dihydroxy-phenyl) pentane-1,5-dione and 7,2',4'-trihydroxyflavanone)^299^, 2',4',6'-trihydroxychalcones^300^, naphthyl chalcones^301^ and chalcone thiosemicarbazide derivatives^302^ have been identified as a new class of tyrosinase inhibitors. Interestingly, the most important factors in the efficacy of a chalcone are the location of the hydroxyl groups on both aromatic rings and the number of these hydroxyls and the presence of a catechol moiety don't correlate with increasing tyrosinase inhibition potency^303^.

##### Aurones

Okombi et al. have identified Z-benzylidenebenzofuran-3(2H)-one and analogs as human tyrosinase inhibitors. However, they found that aurones are weak inhibitors, but their derivatives with two or three hydroxyl groups preferably at 4,6 and 4' positions make them significant tyrosinase inhibitors. For example, the most potent aurone, 4,6,4'-trihydroxyaurone induces 75% inhibition at 0.1 mM concentration and is highly effective compared to kojic acid^304^. In addition to synthetic compounds, several natural compounds such as (2'R)-2',3'-dihydro-2'-(1-hydroxy-1-methylethyl)-2,6'-bibenzofuran-6,4'-diol^305^ and 2-arylbenzofurans isolated from *Morus notabilis*^306^ and *Morus yunnanensis*^230^, benzofuran flavonoids such as mulberrofuran G (MG) and albanol B (AB) isolated from *Morus* sp^307^ and macrourins E isolated from *Morus macroura* (IC_50_ = 0.39 µM) are potent tyrosinase inhibitors among aurones^308^.

#### Phenolic acids

Phenolic acids are divided into hydroxybenzoates and hydoxycinnamates. The most common hydroxycinnamates are *p*-coumaric, caffeic and ferulic acids. So far, *p*-hydroxybenzoic acid, chlorogenic acid (the ester of caffeic *acid*), vanilic acid (4-hydroxy-3-methoxybenzoic acid) and protocatechuic acid (a dihydroxybenzoic acid) from *Hypericum laricifolium* Juss^154^, protocatechualdehyde (IC_50_ = 0.40 µg/mL) from *Phellinus linteus*^175^, benzoic acid propyl gallate^309^, orsellinic acid (2,4-dihydroxy-6-methylbenzoic acid) and orsellinates (2,4-dihydroxy-6-methyl benzoates)^310^, *p*-coumaric acid from *ginseng* leaves^311^, *m*-coumaric acid^312^, *p*-coumarate^313^ and its derivatives from leaves of *Breynia officinalis*^184^ caffeic acid and its *n*-nonyl ester^314^, ferulic acid from *Spiranthes sinensis*^224^, 4-Hydroxy cinnamic acid^315^, synthetic hydroxycinnamoyl phenylalanyl/prolyl hydroxamic acid derivatives^316^, and seven hydroxycinnamoyl derivatives in green coffee beans^317^ have been investigated for their tyrosinase inhibition activity. Among these, propyl gallate is a reversible and mixed-type inhibitor on diphenolase activity of tyrosinase with *K*_IS_ = 2.135 mM and *K_i_* = 0.661 mM^309^. Furthermore, *n*-butyl, *iso*-propyl, *sec*-butyl, *n*-pentyl, *n*-hexyl and *n*-octyl orsellinates (uncompetitive, with an inhibition constant of 0.99 mM) behaved as inhibitors at 0.50 mM, whereas methyl, ethyl, *n*-propyl, *tert*-butyl, and *n*-cetyl orsellinates acted as tyrosinase activators. Thus, tyrosinase inhibition increased with chain elongation, suggesting that the enzyme site can accept an eight-carbon alkyl chain^310^.

In addition to these compounds, 3-phenylbenzoic acid (3-PBA) was revealed to be the most potent inhibitor against monophenolase (noncompetitive, IC_50_ = 6.97 µM) and diphenolase (mixed type inhibition, IC_50_ = 36.3 µM) activity of mushroom tyrosinase. Also, Oyama et al. have found that some modification such as esterification can abrogate this inhibitory activity of tyrosinase^318^.

#### Stillbenes

**Figure 8:**
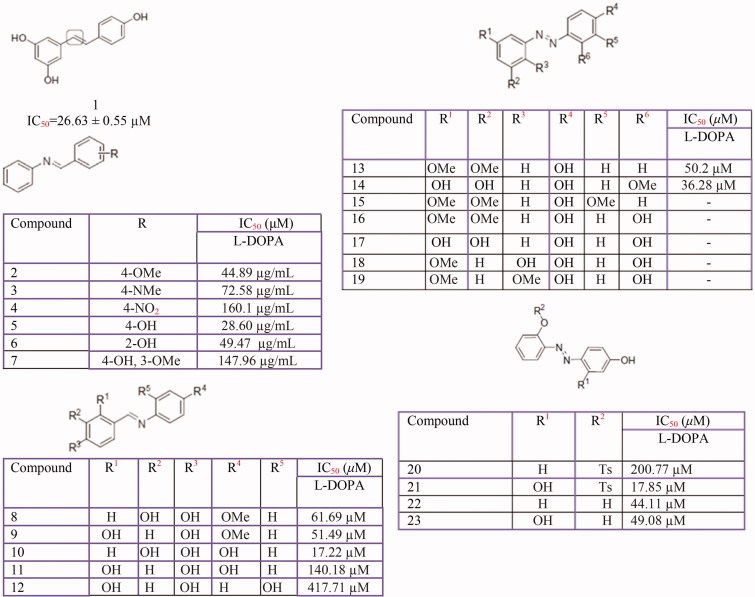
Resveratrol (3,5,4-trihydroxy-trans-stilbene) **(1)**, and its analogs **(2–23)**.

Resveratrol is the most common stilbene. Several stillbenes derivatives from natural and synthetic sources (Figure 8) have been investigated for their tyrosinase inhibition activity including: resveratrol from *Morus alba*^319^, *Pleurotus ferulae*^135^, *vitis viniferae caulis*^320^, *Carignan* grape juice^321^*Artocarpus gomezianus*^322^ and *Streptomyces avermitilis* MA4680^323^ and also, its derivatives fro*m* Dipterocarpaceae plants^324^ and synthetic sources^325^, oxyresveratrol^326^ from *Morus australis*^327^, *Morus alba* L (IC_50_ = 0.10 ± 0.01 µM)^249^ and *Cudrania cochinchinensis* (IC_50_ = 2.33 µM)^255^, azo-resveratrol and its derivatives such as (E)-2-((2,4-dihydroxyphenyl)diazenyl) phenyl 4 methylbenzenesulfonate^328^ and *azo*-oxyresveratrol^329^, *trans*-resveratrol from *Streptomyces avermitilis* MA4680 ^313^, a resveratrol dimer named gnetin C, from melinjo *(Gnetum gnemon*)^330^. Also, several hydroxystillbene compounds from synthetic and semisynthetic sources^331^^,^^332^ and from the extract of *Veratrum patulum*^333^, along with synthetic glycosides of resveratrol, pterostilbene, and pinostilbene^334^, synthetic trans-stilbene derivatives^335^, azastilbene analogs^336^, a newly synthesised stillbene 5-(6-hydroxy-2-naphthyl)-1,2,3-benzenetriol^337^, coumarin-resveratrol hybrids^290^, synthetic polyphenolic deoxybenzoins^218^, hydroxy substituted 2-phenyl-naphthalenes^338^ and 4-(6-hydroxy-2-naphthyl)-1,3-bezendiol^339^ have been studied for their inhibition activity against tyrosinase. However, based on the enzymatic assays, resveratrol did not inhibit the diphenolase activity of tyrosinase, but L-tyrosine oxidation by tyrosinase was suppressed in presence of 100 µM resveratrol. Interestingly, after the 30 min of preincubation of tyrosinase and resveratrol, both monophenolase and diphenolase activities of tyrosinase were significantly suppressed. Furthermore, this effect was reduced with the addition of l-cysteine, which indicated suicide inhibition mechanism of resveratrol^340^. Also, oxyresveratrol^201^ is identified as a tyrosinase substrate like hydroquinone, arbutin, caffeic acid and some other inhibitors. In addition to these studies on resveratrol, Fachinetti et al., have demonstrated that the incorporation of resveratrol into nanostructured lipid carriers allowed an enhanced tyrosinase inhibitory activity^341^.

#### Lignans

Lignans are complex and diverse structures, which are formed from three primary precursors. So far, lignans and lignan glycosides isolated from exocarp of *Castanea henryi*^342^, *Marrubium velutinum* and *Marrubium cylleneum*^343^, *Pinellia ternate*^344^ and *Crataegus pinnatifida*^345^ have been evaluated for their tyrosinase inhibitory potentials. However, these compounds mostly displayed a moderate mushroom tyrosinase inhibitory activity.

### Terpenoid derivatives

Carvacrol is a monoterpenoid phenol. To date, some carvacrol derivatives^346^ from synthetic sources, bakuchiol, a terpene phenol from *Psoralea corylifolia*^21^, iridoid glucosides (another type of monoterpenoids) from *Wulfenia carinthiaca* Jacq^347^ and two new bis-iridoids, namely 7-O-caffeoyl-sylvestroside I and 7-O-(p-coumaroyl)-sylvestroside I isolated from *Scabiosa stellata*^348^ have been investigated for their anti-tyrosinase activities. Among these terpenoid derivatives, Cheng et al. have demonstrated that bakuchiol is a potent inhibitor by applying capillary electrophoresis with reliable online immobilised enzyme microreactor^21^. Also, carvacrol derivatives such as 2-[2-methyl-5-(propan-2-yl)phenoxy]-2-oxoethyl(2E)-3–(2,4-dihydroxyphenyl)prop-2-enoate showed excellent tyrosinase inhibitory activity by a noncompetitive manner with K_i_ value 0.05 µM and IC_50_ = 0.0167 µM^349^.

### Quinone derivatives

The quinones are a class of small molecules that are mostly derived from aromatic compounds such as benzene or naphthalene. Among these compounds, Aloin, an anthraquinone-C-glycoside from *Aloe vera*^349^, anthraquinones from *Polygonum cuspidatum*^350^ and tanshinone IIA (IC_50_ = 1214 *µ*M) have been verified as tyrosinase inhibitors^239^.

### Phenyl derivatives

**Figure 9:**
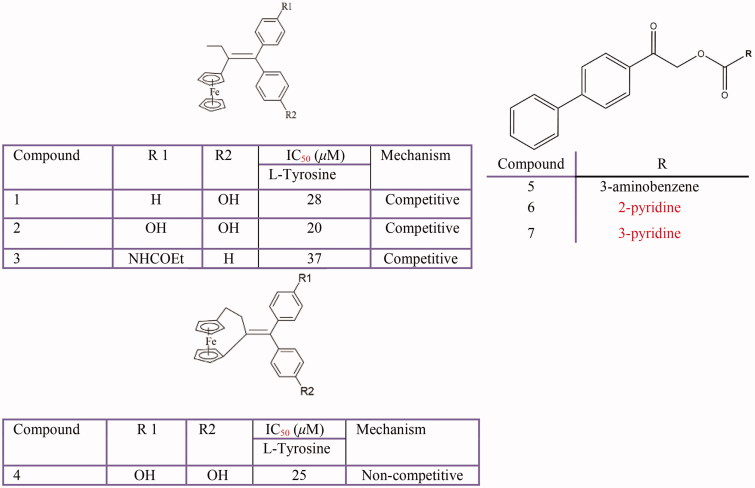
Some phenyl derivatives: aryl butane **(1–4)**, biphenyle ester **(5–7)**.

Several biphenyl derivatives^351^ (Figure 9) such as 4,4'-dihydroxybiphenyl^352^, biphenyl ester derivatives^340^, biphenyl construction from flavan-3-ol substrates^353^, hydroxylated biphenyls^26^, functionalised bis-biphenyl substituted thiazolidinones^36^, phenylbenzoic acid derivatives^354^, phenylethylamide and phenylmethylamide derivatives^355^, hydroxy substituted 2-phenyl-naphthalenes^318^, 4-hydroxyphenyl beta-d-oligoxylosides^356^, benzenethiol or phenylthiol^357^, 2-((1Z)-(2–(2,4-dinitrophenyl)hydrazin-1-ylidene)methyl) phenol^358^ and 4-[(4-hydroxyphenyl)azo]-benzenesulfonamide^359^, have been identified as tyrosinase inhibitors.

### Pyridine, Piperidine, pyridinones and hydroxypyridinone derivatives

**Figure 10:**
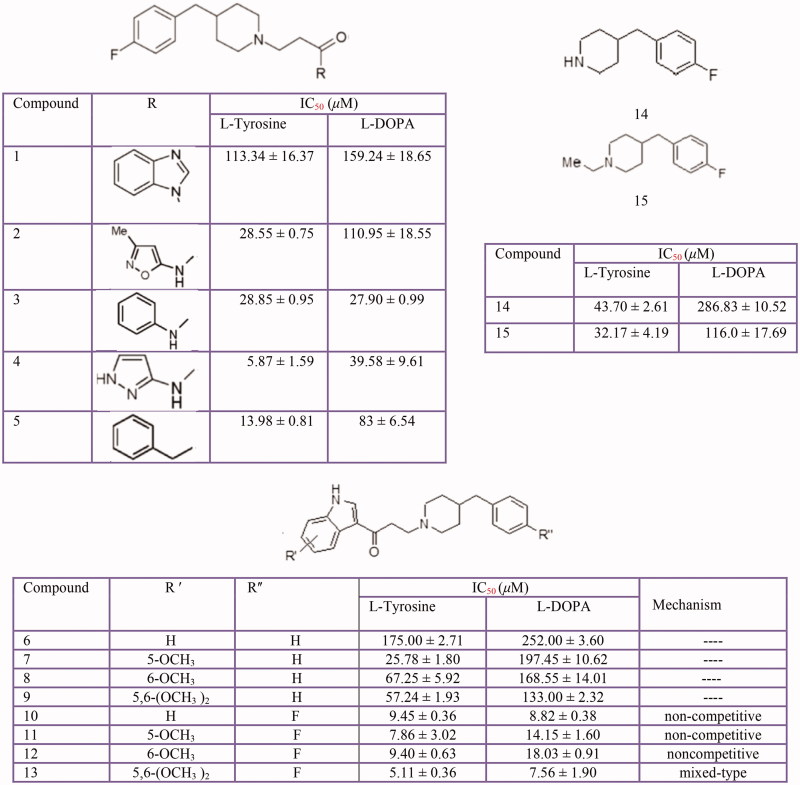
Inhibitory effects of some piperidine derivatives on mushroom tyrosinase activity. 4–(4-fluorobenzyl) piperidine derivatives **(1–5**) indole derivatives **(6–13)** amine **(14)** and N-ethyl **(15)**.

Some hydroxypyridinone derivatives^360^, 3-hydroxypyridine-4-one derivatives^361^ hydroxypyridinone-L-phenylalanine^362^ and pyridinones^363^ have been characterised for their antityrosinase activity (Figure 10). Among these inhibitors, one mixed-type inhibitor from hydroxypyridinone-l-phenylalanine conjugates named ((S)-(5-(benzyloxy)-1-octyl-4-oxo-1,4-dihydropyridin-2-yl) methyl 2-amino-3-phenylpropanoate) showed potent inhibitory effect with IC_50_ values of 12.6 and 4.0 µM for monophenolase and diphenolase activities, respectively^362^.

### Thiosemicarbazones, Thiosemicarbazide and other Thio derivatives

**Figure 11:**
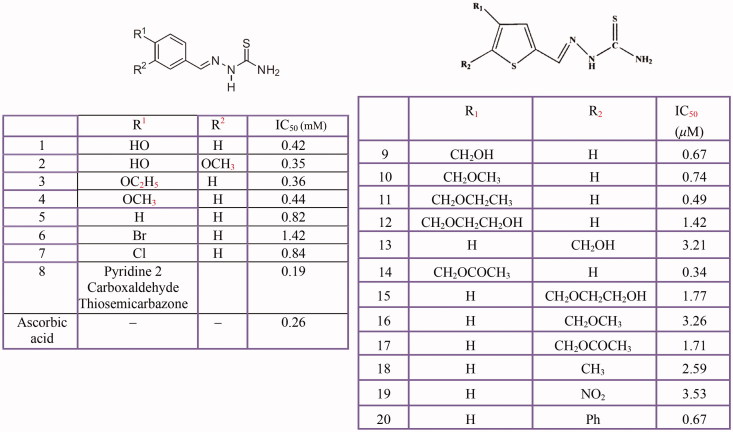
Inhibitory effects of some thiosemicarbazone derivatives on the tyrosinase monophenolase activity.

Several kinds of thiosemicarbazone derivatives^38^^,^^34^^,^^364–376^ has been investigated as possible tyrosinase inhibitors (Figure 11). Furthermore, some benzaldehyde derivatives of thiosemicarbazone such as chlorobenzaldehyde thiosemicarbazones^363^, *p*-hydroxy and *p*-methoxy benzaldehyde thiosemicarbazone^362^ along with *p*-methoxybenzaldehyde thiosemicarbazone and 4-dimethylaminobenzaldehyde-thiosemicarbazone and 4-dimethylaminobenzaldehyde-N-phenyl-thiosemicarbazone^377^ were evaluated for their inhibitory activities on mushroom tyrosinase.

Based on the findings, the appropriate functionalisation of thiosemicarbazone may be improved the inhibitory activity of these inhibitors. Dong et al. believe that the sterically bulky group at the C-4 position of the thiophene ring contributes to this activity. For example, the 4-functionalisation thiophene-2-carbaldehyde thiosemicarbazone with a methoxyacetyl group^368^ or introducing benzene ring to the 4-functionalised ester group^367^ enhanced inhibitory activity of thiophene-2-carbaldehyde thiosemicarbazone. However, 5-functionalisation decreased its inhibitory activity. Also, Soares et al., have demonstrated thiosemicarbazones Thio-1, Thio-2, Thio-3 and Thio-4 substituted with oxygenate moieties, displayed better inhibitory activity (IC_50_ 0.42, 0.35, 0.36 and 0.44 mM, respectively) than Thio-5, Thio-6, Thio-7 and Thio-8^34^.

**Figure 12:**
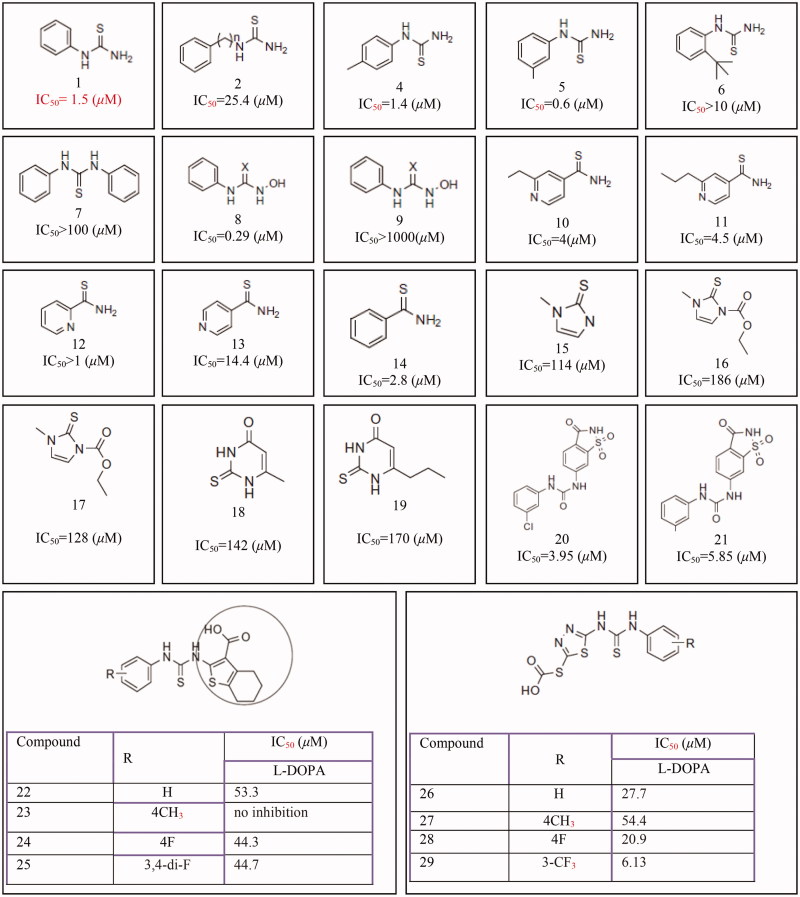
Thiourea derivatives **(1–14),** methimazole **(15)**, carbimazole **(16),** thiouracil **(17)**, methylthiouracil **(18)**, propylthiouracil **(19)**, 6–(3-chlorophenylurenyl) saccharin **(20)**, 6–(3-iodophenylthiourenyl) saccharin **(21)**, 4,5,6,7-tetrahydro- 2-[[(phenylamino)thioxomethyl]amino]-benzo[b]thiophene-3-carboxylic acid derivatives **(22–25)**, 2–(1,3,4-thiadiazol-2-yl) thio acetic acid derivatives **(26–29)**.

In addition to thiosemicarbazone derivatives, thiosemicarbazide and its derivatives^378–381^, 5-benzylidene(thio)barbiturate-beta-d-glycosides^382^, *n*-alkyl^383^, *p*-phenylene-bis, phenyl^384^, benzyl, *p*-xylidine-bis and *p*-pyridine dithiocarbamate sodium salts^385^, diethyldithiocarbamate, phenylthiourea^3^^86^ and other thiourea derivatives (Figure 12) such as methimazole, thiouracil, methylthiouracil, propylthiouracil, ambazone, and thioacetazone^387^ have been identified as tyrosinase inhibitors.

### Azole and thiazolidine derivatives

**Figure 13:**
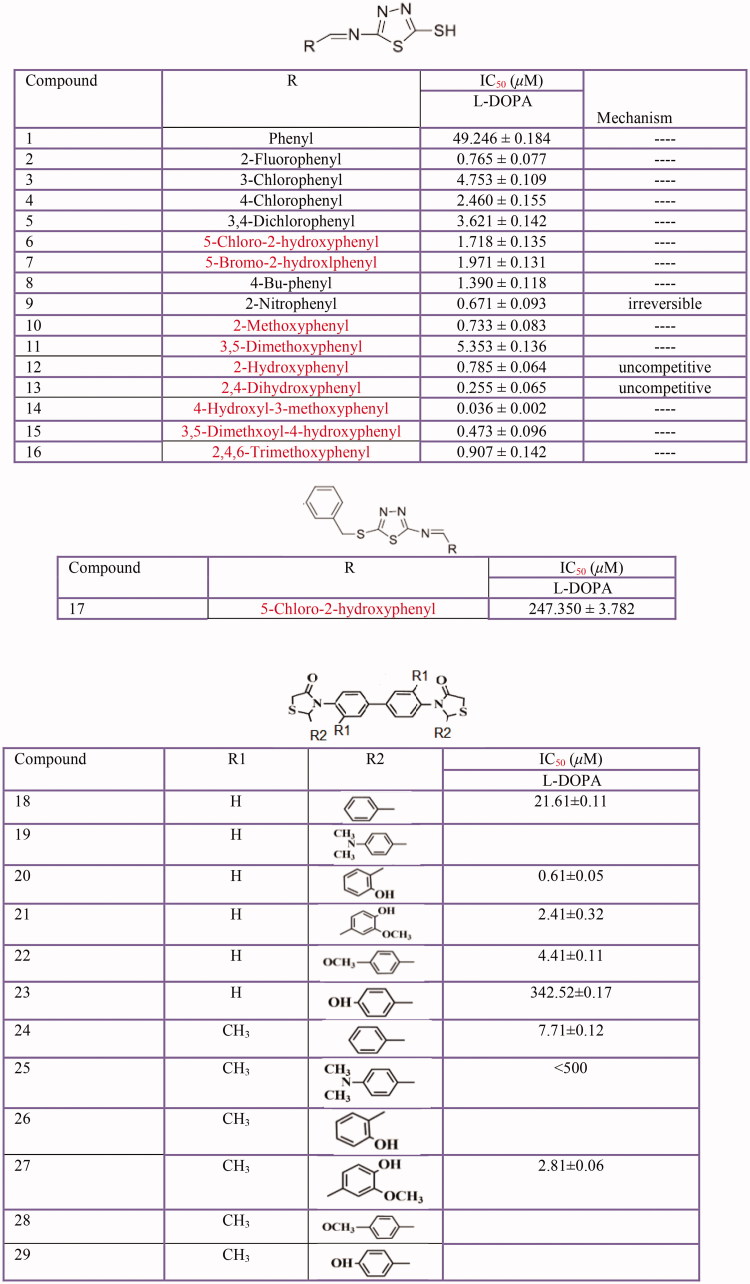
Thiadiazole derivatives: 1,3,4-thiadiazole derivatives **(1–17)** and thiazolidinones derivative **(18–29)**.

So far, several azole derivatives (Figure 13) have been studied for their tyrosinase inhibitory activity^388^. The discovered new types of inhibitors included DL-3(5-benzazolyl) alanines and alpha-methyldopa analogs^389^, aryl pyrazoles^390^, heterocyclic hybrids based on pyrazole and thiazolidinone scaffolds^391^, 3,5-diaryl-4,5-dihydro-1H^392^ and 3,5-diaryl pyrazole derivatives^393^, pyrazolo[4,3-e][1,2,4]triazine sulfonamides and sildenafil^394–396^, 1,3-oxazine-tetrazole^397^, indole-spliced thiadiazole^398^, benzimidazole-1,2,3-triazole hybrids^399^, 1,2,3-triazole-linked coumarinopyrazole conjugates^400^, isoxazolone derivatives^401^ 5(4H)-oxazolone derivative^402^, imidazolium ionic liquids^403^, thiazolyl resorcinols^404^ have demonstrated the inhibitory effect on tyrosinase. Furthermore, some thiazolidine derivatives have been evaluated for their tyrosinase inhibitory activity including azo-hydrazone tautomeric dyes substituted by thiazolidinone moiety^405^, (Z)-5-(2,4-dihydroxybenzylidene) thiazolidine-2,4-dione^406^, 5-(substituted benzylidene) thiazolidine-2,4-dione derivatives^407^, (2RS,4R)-2-(2,4-dihydroxyphenyl)thiazolidine-4-carboxylic acid^408^, 2-(substituted phenyl) thiazolidine-4-carboxylic acid derivatives^409^ and (Z)-5-(3-hydroxy-4-methoxybenzylidene)-2-iminothiazolidin-4-one^410^.

### Kojic acid analogs

Kojic acid is a well-known tyrosinase inhibitor. When DL-DOPA, norepinephrine and dopamine are oxidised by tyrosinase, Kojic acid inhibits effectively the rate of formation of pigmented product(s) and of oxygen uptake^411^. Furthermore, several of its derivatives have demonstrated a potent tyrosinase inhibitory activity^361^^,^^412–418^. Noh et al. have modified kojic acid with amino acids and screened their tyrosinase inhibitory activity. Among them, kojic acid-phenylalanine amide showed a strong noncompetitive inhibition^417^. Interestingly, some kojic acid derivatives despite their depigmenting activities did not display tyrosinase inhibitory activitiy^419^.

**Figure 14:**
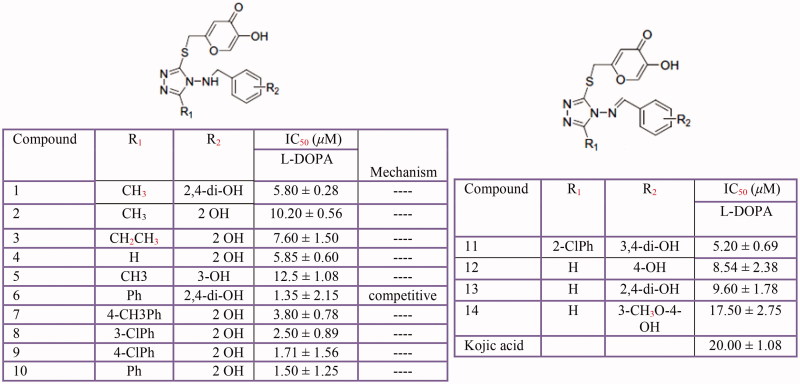
Some kojic acid analogs: hydroxybenzaldehydebased kojic acid analogs (5-substituted-3-[5-hydroxy-4-pyrone-2-ylmethylmercapto]-4-arylmethylamino-1,2,4-triazole **(1–10)** and 5-substituted-3-[5-hydroxy-4-pyrone-2-yl-methylmercapto]-4-arylmethyleneamino-1,2,4-triazole **(11–14)**.

Recently, Xie et al. have reported a kojic acid analog namely 5-phenyl-3-[5-hydroxy-4-pyrone-2-yl-methylmercapto]-4-(2,4-dihydroxylbenzylamino)-1,2,4-triazol as a potent competitive tyrosinase inhibitor with an IC_50_ value of 1.35 ± 2.15 µM^412^. Tyrosinase inhibitory activity of some kojic acid derivatives is shown in Figure 14.

### Benzaldehyde derivatives

**Figure 15:**
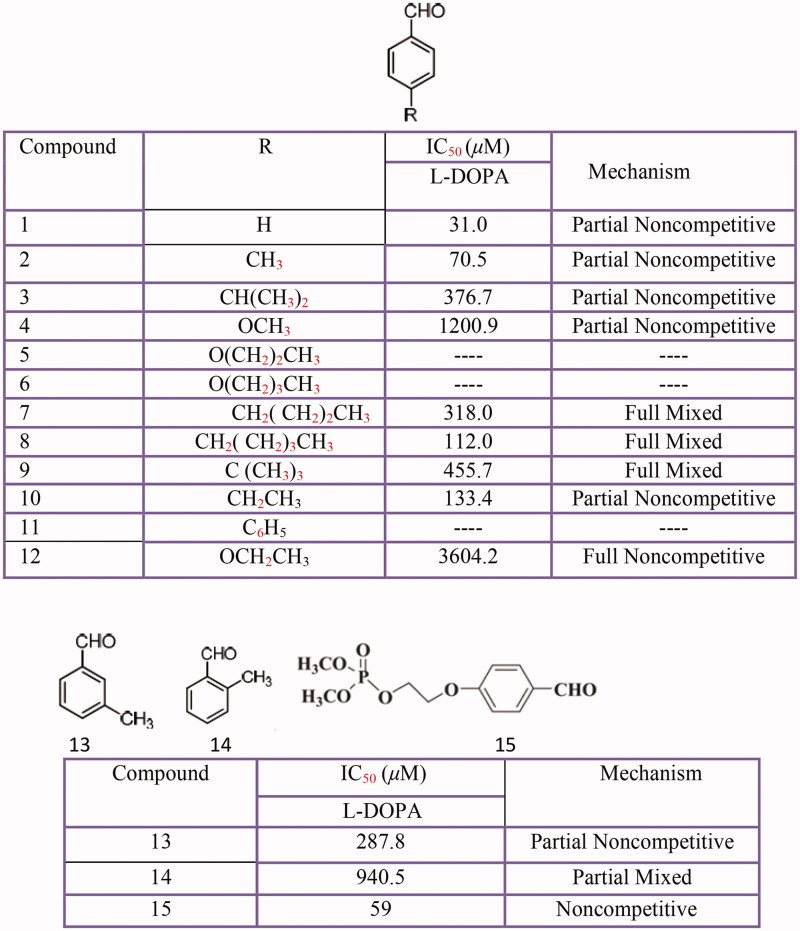
Benzaldehyde derivatives: 4-substituted benzaldehyde **(1–15).**

Benzaldehyde^420^ and its derivatives^421^, hydroxy- or methoxy-substituted benzaldoximes and benzaldehyde-O-alkyloximes^422^, piperonal or 4-(methylenedioxy) benzaldehyde mesoionic derivatives^423^, 4-hydroxybenzaldehyde derivatives^424^, anisaldehyde^425^ have been investigated for their inhibitory activities against tyrosinase (Figure 15).

Among these derivatives, 3,4-dihydroxybenzaldehyde-*O-*ethyloxime (IC_50_ = 0.3 ± 0.1 µM) is of the same magnitude as one of the best tyrosinase known inhibitors tropolone (IC_50_ = 0.13 ± 0.08 µM)^422^. However, in benzaldehyde derivatives, the presence of the aldehyde group and the terminal methoxy group in C4 was found to play an important role in its inhibitory effect. But, due to their lower activity levels or serious side effects, unfortunately, most 4-substituted benzaldehyde derivatives cannot be considered for practical use^421^.

### Carboxylic acids

Inhibitory effects of pyruvic acid, acrylic acid, propanoic acid, 2-oxo-butanoic acid, and 2-oxo-octanoic acid^124^, (S)- and (R)-6-hydroxy-2,5,7,8-tetramethylchroman-2-carboxylic acids^426^ have been investigated on tyrosinase activity.

Based on the findings investigated by Gheibi et al., aliphatic carboxylic acids have dual effects on the monophenolase and diphenolase activities of mushroom tyrosinase. They have found that optimal diphenolase activity of tyrosinase takes place in the presence of *n*-alkyl acids (pyruvic acid, acrylic acid, propanoic acid, 2-oxo-butanoic acid, and 2-oxo-octanoic acid). While, the monophenolase activity is inhibited by all types of *n*-alkyl acids. They have believed that there is a physical difference in the docking of *mono*- and *o*-diphenols to the tyrosinase active site. On the other hand, the binding of acids occurs through their carboxylate group with one copper ion of the binuclear site. So these carboxylic acid compounds completely block the monophenolase reaction, by preventing monophenol binding to the oxyform of the enzyme^124^.

### Xanthate derivative

The inhibitory effect of some synthesised xanthates including C_3_H_7_OCS_2_Na, C_4_H_9_OCS_2_Na, C_5_H_11_OCS_2_Na, C_2_H_5_OCS_2_Na, and C_6_H_13_OCS_2_Na have been examined for inhibition of both monophenolase and diphenolase activities of mushroom tyrosinase. Based on the reports, C_3_H_7_OCS_2_Na and C_4_H_9_OCS_2_Na showed a mixed inhibition pattern on monophenolase activity but C_5_H_11_OCS_2_Na and C_6_H_13_OCS_2_Na showed a competitive and C_2_H_5_OCS_2_Na showed uncompetitive inhibition pattern. For diphenolase activity, C_3_H_7_OCS_2_Na and C_2_H_5_OCS_2_Na showed mixed inhibition but C_4_H_9_OCS_2_Na and C_5_H_11_OCS_2_Na and C_6_H_13_OCS_2_Na showed competitive inhibition^427^. According to their results, it seems that the lengthening of the hydrophobic tail of the xanthates leads to a decrease of the *K_i_* values for monophenolase inhibition and an increase of the *K_i_* values for diphenolase inhibition^428^.

## Other tyrosinase inhibitors

Except the inhibitors listed above, other compounds have also been registered for their tyrosinase inhibitory activity by different researchers such as: two Keggin-type polyoxometalates containing glycine as potent inorganic reversible inhibitors^429^, cadmium ions with an IC_50_ of 2.92 ± 0.16 mM^48^ and rifampicin with an IC_50_ = 90 ± 0.6 µM^9^ as reversible and noncompetitive inhibitors, ammonium tetrathiotungstate^430^, amoxicillin (IC_50_ = 9.0 ± 1.8 mM)^431^, mallotophilippen A and B ^432^ α-naphthol and β-naphthol^433^, red koji extracts (IC_50_ of 5.57 mg/mL)^434^ and alpha-hydrazinophloretic acid^435^ as competitive inhibitors and rottlerin as a mixed inhibitor^432^. Furthermore, *n*-alkyl sulfates^436^, sericin extracted from tasar silk fiber waste^437^, 2-hydroxy-3-methylcyclopent-2-enone (IC_50_ = 721.91 µg mL^−1^) isolated from ribose-histidine Maillard reaction products^438^, three natural compounds from safflower^439^ and mimosine^386^ and ethylenediamine^440^ are other kinds of tyrosinase inhibitors.

## Synergistic effects of tyrosinase inhibitors

Synergistic strategy for tyrosinase inhibitors is a useful strategy for the improvement of their inhibitory activities. Based on the findings, the mixtures of glabridin:resveratrol, glabridin:oxyresveratrol, resveratrol:oxyresveratrol, phenylethylresorcinol:resveratrol^441^, oxyresveratrol:dioscin^442^, aloesin:arbutin^443^, 4-methyl catechol: catechol^444^, 3-(2,4-dihydroxyphenyl)propionic acid:l-ascorbic acid^445^, dihydromyricetin:vitamin D3^37^, linderanolide B combined with arbutin, 1-phenyl-2-thiourea or kojic acid^446^, have shown synergistic effect on tyrosinase. These studies may provide a scientific strategy for screening effective tyrosinase inhibitors.

## Conclusion

Due to the vital role of tyrosinase in the enzymatic browning of food and depigmentation disorders in humans, its inhibitors have been considered by researchers, extensively. As mentioned above, natural sources such as plants and microorganisms and their effective compounds have wonderful potential as organic anti-tyrosinase sources.

However, the majority of the compounds identified from natural sources were isolated from plants but, recently, microorganisms are considered as potential sources of tyrosinase inhibitors. It is interesting that despite the diversity of natural inhibitors, a large number of tyrosinase inhibitors are phenolic-based structures. Many researchers have designed appropriate scaffold inspired by the structure of natural compounds and developed novel synthetic inhibitors. In this paper, many natural, semi-synthetic and synthetic inhibitors have been summarised and the inhibitory effects of these compounds on the tyrosinase activity are discussed.

Based on the results, phenolic compounds (simple phenols and polyphenols) and their derivatives and several compounds including terpenoid, phenyl, pyridine, piperidine, pyridinone, hydroxypyridinone, thiosemicarbazone, thiosemicarbazide, azole, thiazolidine, kojic acid, benzaldehyde and xanthate derivatives were characterised as potent tyrosinase inhibitors. The appropriate functionalisation of these inhibitors such as C-6 and C-7 hydroxyl groups of the isoflavone skeleton, 4-functionalisation thiophene-2-carbaldehyde thiosemicarbazone with a methoxyacetyl group and the aldehyde group and methoxy group in C4 of benzaldehyde derivatives may be improved the inhibitory activity of these inhibitors. Furthermore, in cholcone derivatives, the location of the hydroxyl groups on both aromatic rings and the number of hydroxyls is an important factor in the efficacy of a chalcone. In contrast, some modifications such as the prenylation or the vinylation of some flavonoid molecules do not enhance their tyrosinase inhibitory activity while deglycosylation of some flavonoid glycosides by far-infrared irradiation can be improved tyrosinase inhibitory activity. Interestingly, among different inhibitors, some compounds, especially hydroquinone and its known derivatives (α and β-arbutin), are described as both a tyrosinase inhibitor and a substrate.

Actually, the main objective of this review is to provide a useful source of effective tyrosinase inhibitors. However, despite the existence of a wide range of tyrosinase inhibitors from natural and synthetic sources, only a few of them, in addition to being effective, are known as safe compounds. Therefore, it is recommended to examine the efficacy and safety of inhibitors by in vivo models, along with *in vitro* and docking experiments, especially for the application of such materials in food and medicinal products. Finally, we hope that the information provided in this study, which is the result of numerous researchers’ efforts, could serve as leads in the search for effective anti-tyrosinase agents from natural and synthetic sources with increased efficiency and safety in the food and cosmetics industries.
